# Ecological and Phytochemical Studies on *Euphorbia retusa* (Forssk.) from Egyptian Habitat

**DOI:** 10.1155/2018/9143683

**Published:** 2018-02-01

**Authors:** Mohamed Shaaban, Mahmoud Ali, Michel Feussi Tala, Abdelaaty Hamed, Amal Zaki Hassan

**Affiliations:** ^1^Chemistry of Natural Compounds Department, Division of Pharmaceutical Industries, National Research Centre, El-Behoos St. 33, Dokki, Cairo 12622, Egypt; ^2^Department of Organic and Biomolecular Chemistry, University of Göttingen, Tammannstrasse 2, 37077 Göttingen, Germany; ^3^Plant Ecology and Range Management Department, Desert Research Centre, Cairo, Egypt; ^4^Organic and Bioorganic Chemistry, Department of Chemistry, Bielefeld University, Universitätsstraße 25, 33501 Bielefeld, Germany

## Abstract

This study deals with the ecology, phytochemistry, and biological activity investigation of *Euphorbia retusa*, belonging to Euphorbiaceae family, obtained from Egypt. Ecologically, *Euphorbia retusa* secretes white sap inhibiting the growth of the other species, so *Euphorbia retusa* is forming complete patches. Phytochemical study of the plant was visualized intensively based on its extraction with a protic organic solvent, working up and purifying its entire bioactive compounds using a series of different chromatographic techniques. A broad range of diverse compounds were isolated, namely, 1-hexacosanol (**1**), 3*β*-hydroxy-24-methylene-9,19-cyclolanostane; 24-methylenecycloartanol (**2**), 3*β*-hydroxy-9,19-cyclolanostane; cyclolaudanol (**3**), 3*β*,24*S*-Ergost-5-en-ol (**4**), and methyllinoleate. Additionally, GC-MS analysis of the unpolar fractions detected the existence of *n*-dodecane, methyllaurate, 6,10,14-trimethyl-pentadecan-2-one (**5**), 6,10-dimethyl-undecan-2-one (**6**), 2-methyl-hexadecanal (**7**), methylpalmitate, methyl-9,12,15-octadecatrienoate (**8**), and *n*-heneicosane (**9**). A full assignment for compounds **2** and **3** using 1 and 2 DNMR was carried out herein for the first time. The antimicrobial activity of the strain extract and obtained compounds was studied using a panel of pathogenic bacterial strains. The in vitro cytotoxicity of the compounds as well as the crude extract was studied against the human cervix carcinoma cell line (KB-3-1).

## 1. Introduction

Euphorbiaceae (spurge family) is a large and polymorphic genus with about 8100 species, out of which 2000 Euphorbia species were found. This family was reported recently to comprise about 300 genus and 10,000 species, which are used in folk medicine against the venomous bites and trichiasis and known as wart remover [1]. Species of Euphorbia are characterized by high ecological amplitudes in tropical, subtropical, and warm temperate regions, and they are widely spread around the world, and distributed mainly in North Africa and in the temperate parts of Asia, but mainly in the Mediterranean region [2–5].

In Egypt, *Euphorbia retusa* is distributed in the Mediterranean coastal belt, Sinai Peninsula, and eastern and western desert especially in sandy and desert wadis and plains forming complete patches maybe due to its allopathic activities. Members of Euphorbiaceae secrete a white milky sap or latex. This sap is of medicinal importance and affects the associated species or prevents the growth of plant species [6]. The western Mediterranean coast of Egypt extended for about 500 km from Alexandria to El-Sallum. The main geomorphological features are coastal wadis and Marmarica Plateau [7]. Sidi Barrani area is a coastal region that belongs to the subarid region with mild winter and arid summer. The annual average temperature is 18.4°C and rainfall is 150 mm [8]. Vegetation of the study area can be recognized as coastal sand dunes dominated with *Ammophila arenaria* and *Euphorbia paralias* and inland areas dominated with *Haloxylon scoparium* and *Asphodelus aestivus* [9–11]. Phytochemically, *Euphorbia retusa* was reported as a rich source of diverse bioactive compounds. Different classes of compounds have been isolated from various parts of *Euphorbia retusa*, of which the main groups are terpenoids and flavonoids [12]. Additionally, topical pharmaceutical compounds comprising a dry extract of Euphorbia species or a drug or a active agent with excipients useful for the treatment of anorectal diseases and colon diseases such as hemorrhoids, fissures, cracks, fistulas, abscesses, and inflammatory bowel disease are provided [13].

In the present study, measuring of the density, frequency, and flowering sequences of *Euphorbia retusa* and its associated species was investigated. Additionally, further studies to identify the secondary metabolites of *Euphorbia retusa* collected from Egyptian habitats and evaluate their antimicrobial activities was presented as well.

## 2. Materials and Methods

### 2.1. General Procedure

NMR spectra were measured on Varian Unity 300 and Varian Inova 500 spectrometers. Electron spray ionization mass spectrometry (ESI-HRMS) was performed by a Finnigan LCQ ion trap mass spectrometer coupled with a Flux Instruments' (Basel, Switzerland) quaternary pump Rheos 4000 and a HP 1100 HPLC (Nucleosil column EC 125/2, 100-5, C 18) with an autosampler (Jasco 851-AS, Jasco Inc., Easton, MD, USA) and a diode array detector (Finnigan Surveyor LC System). High-resolution mass spectra (HRMS) were recorded by ESI-MS on an Apex IV 7 Tesla Fourier-transform ion cyclotron resonance mass spectrometer (Bruker Daltonics, Billerica, MA, USA). GC-MS was performed by a Trace GC-MS Thermo Finnigan, ionization mode EI eV 70, instrument equipped with a capillary column CP-Sil 8 CB for amines (length: 30 m; inside diameter: 0.25 mm; outside diameter: 0.35 mm; and film thickness: 0.25 *µ*m). The analysis was carried out at programmed temperatures with initial temperature 40°C (kept for 1 min) which is then increased at a rate of 10°C/min to reach the final temperature 280°C (kept for 10 min), injector temperature was 250°C, and detector (mode of ionization: EI) temperature was 250°C, with He as a carrier gas at a flow rate of 1 mL/min, a total run time of 27 min, and an injection volume of 0.2 *µ*L. Characterization of individual unpolar compounds by GC-MS was performed in triplicate. *R*_*f*_ values were determined on Polygram SIL G/UV_254_ (Macherey & Nagel, Düren, Germany). Size exclusion chromatography was performed on Sephadex LH-20 (Lipophilic Sephadex; Amersham Biosciences, Ltd., purchased from Sigma-Aldrich Chemie, Steinheim, Germany).

### 2.2. Plant Material

The plant samples were collected during the spring of 2014 from 5 homogeneous sites in their floristic composition, but they are different in the habitats (Table 1). Site locations were recorded by using 12 channels (German GPS). The samples were shade-dried and grinded by an electrical mill mesh. A voucher specimen of the plant was identified and authenticated in the Laboratory of Botany at the Desert Research Centre, Egypt.

### 2.3. Preparation of the Plant Crude Extract

250 g of the powdered whole plant parts was macerated (24 hrs) three times with 1.5 L of methanol at room temperature. The supernatant was filtered using a Whatman filter paper and concentrated in vacuo till dryness yielding 36.63 g of a dark green crude extract.

### 2.4. Isolation and Purification of the Plant Compounds

The afforded plant crude extract (36.63 g) was separated by column chromatography on silica gel (100 × 5 cm) using *n*-hexane-CH_2_Cl_2_-MeOH gradient (0.5 L hexane, 0.5 L hexane-CH_2_Cl_2_ [90 : 10], 0.5 L hexane-CH_2_Cl_2_ [80 : 20], 0.5 L hexane-CH_2_Cl_2_ [50 : 50], 1 L CH_2_Cl_2_, 0.5 L CH_2_Cl_2_-MeOH [97 : 3], 0.5 L CH_2_Cl_2_-MeOH [95 : 5], 1 L CH_2_Cl_2_-MeOH [90 : 10], 0.5 L CH_2_Cl_2_-MeOH [50 : 50]; 0.5 L MeOH). According to thin layer chromatography (TLC) monitoring using different eluent systems with different polarities (hexane-CH_2_Cl_2_ [90 : 10; 80 : 20, 50 : 50]; CH_2_Cl_2_, CH_2_Cl_2_-MeOH [98 : 2], CH_2_Cl_2_-MeOH [95 : 5], CH_2_Cl_2_-MeOH [93 : 7], CH_2_Cl_2_-MeOH [90 : 10]) visualized by UV and then spraying with anisaldehyde/sulphuric acid and heating, five fractions were obtained: FI (3.6 g), FII (5.23 g), FIII (7.2 g), FIV (7.7 g), and FV (8.2 g). Fractions III–V showed similarity in their containing of a major UV nonabsorbing band, which was detected as intensive pink-violet on spraying with anisaldehyde/sulphuric acid. Purification of this band using a silica gel column eluted with cyclohexane afforded two colorless solids of 1-hexacosanol (**1**, 200 mg) and 24-methylenecycloartanol (**2**, 2.11 g). An increase in the polarity of the eluent system by gradual addition of CH_2_Cl_2_ to cyclohexane afforded 3*β*-hydroxy-9,19-cyclolanostane (**3**, 6 mg) and 3*β*,24*S*-Ergost-5-en-ol (**4**, 20 mg) as colorless solids, in addition to a colorless oil of methyllinoleate (25 mg). An application of the unpolar fractions to I-II to GC-MS analysis detected the existence of further nine compounds (Table 2).

### 2.5. Biological Activities

#### 2.5.1. Antimicrobial Assay

Antimicrobial assays using the agar diffusion test were performed as described previously [14]. Origin of test strains: *M. miehei* Tü 284 and *Streptomyces viridochromogenes* Tü 57 were obtained from the collection of H. Zähner (University of Tübingen, Germany), and *Chlorella vulgaris* was provided by the algal collection from Göttingen. *B. subtilis* ATCC 6051 was obtained from the American Type Culture Collection, while *S. aureus*, *E. coli*, and *C. albicans* are clinical isolates from Göttingen hospitals. Strains are kept in the strain collection of H. Laatsch, Institute of Organic and Biomolecular Chemistry, Georg-August University, Göttingen, Germany. Ciprofloxacin and erythromycin were used as reference antibiotics for Gram-positive and -negative bacteria testing, while nystatin was served as a reference antimycotic agent for fungi, yeasts, and algae.

#### 2.5.2. Antibacterial Assay

Antibacterial activity testing of the crude extract of *Euphorbia retusa* extract and the isolated pure compounds was further carried out against a wide panel of test microorganisms comprising Gram-positive bacteria (*Bacillus subtilis* DSMZ 704, *Micrococcus luteus* DSMZ 1605, and *Staphylococcus warneri* DSMZ 20036) and Gram-negative bacteria (*Escherichia coli* DSMZ 1058 and *Pseudomonas agarici* DSMZ 11810). Paper-disk diffusion assay [15] has been performed. 20 mL of the medium seeded with test organisms was poured into 9 cm sterile Petri dishes. After solidification, the paper disks (6 mm diameter) were placed on inoculated agar plates and allowed to diffuse the loaded substances into refrigerator at 4°C for 2 h. The plates were incubated for 24 h at 35°C. Bacteria were grown on the nutrient agar medium: 3 gL^−1^ beef extract, 10 gL^−1^ peptone, and 20 gL^−1^ agar. The pH was adjusted to 7.2. After incubation, the diameters of inhibition zones were measured.

#### 2.5.3. Cytotoxicity Assay

The KB-3-1 cells were cultivated as a monolayer in DMEM (Dulbecco's modified Eagle medium) with glucose (4.5 gL^−1^), l-glutamine, sodium pyruvate, and phenol red, supplemented with 10% (KB-3-1) foetal bovine serum (FBS). The cells were maintained at 37°C and 5.3% CO_2_-humidified air. On the day before the test, the cells (70% confluence) were detached with trypsin-ethylenediamine tetraacetic acid solution (0.05%; 0.02% in DPBS) and placed in sterile 96-well plates in a density of 10,000 cells in 100 *μ*L medium per well. The dilution series of the compounds were prepared from stock solutions in DMSO of concentrations of 100 mm, 50 mm, or 25 mm. The stock solutions were diluted with culture medium (10% FBS) down to the picomolar (pM) range. The dilution prepared from the stock solution was added to the wells. Each concentration was tested in six replicates. Dilution series were prepared by pipetting the liquid from well to well. The control contained the same concentration of DMSO as the first dilution. After incubation for 72 h at 37°C and 5.3% CO_2_-humidified air, 30 *μ*L of an aqueous resazurin solution (175 *μ*M) was added to each well. The cells were incubated under the same conditions for 5 h. Subsequently, the fluorescence was measured. The excitation was measured at a wavelength of 530 nm, whereas the emission was recorded at a wavelength of 588 nm. The IC_50_ values were calculated as a sigmoidal dose-response curve using GRAPHPADPRISM 4.03. The IC_50_ values equal the drug concentrations, at which vitality is 50% [16, 17].

## 3. Results and Discussion

### 3. 1. Ecological Study

*Euphorbia retusa* is a glabrous glaucous perennial species, with high range (20–60 cm), sometimes flowering in the first year, and stems erect, many from a woody base. Leaves are sessile, and the cauline is 1–5 × 0.3–0.6 cm oblong-linear, alternate, and base-rounded. Apex is acute to retuse. Margin is acutely serrate and umbrella-like, and floral leaves are opposite. Cyanthia are 2.5–3 mm long in forked umbels.

The data shown in Table 1 represent 5 sites which were selected for field studies. These sites are located about 30 km west of Sidi Barrani city, comprising 4 plant communities and three habitats, namely, plateau, slopes, and desert plains. Selection of the sites was depending on habitat heterogeneity and the difference of floristic composition along transect from the north to south for about 25 km. The study area has been extensively used for barely cultivation and grazing of livestock. Apart from that, *Euphorbia retusa* is neither grazed nor eaten by humans, and its patches remain abundant inside the pasture land.

### 3.2. Vegetation

A total of 30 species (22 perennials and 8 annuals) were recorded in the study area (Table 3). The richest sites are 2, 3, and 4 with 15 species per site, whereas site 1 is the poorest site in vegetation. The mean of species per site is 14.4 ± 0.89.

Besides *Euphorbia retusa* species, there are three species presented in all sites, namely, *Thymelaea hirsuta* and *Haloxylon scoparium* as perennials and *Medicago intertexta* as annuals. They followed with another medicinal plant (*Asphodelus aestivus*) with frequency (80%). Site (1) occupies the inland plateau and is dominated by *Haloxylon scoparium* followed by *Asphodelus aestivus*, *Deverra tortuosa*, and *Euphorbia retusa*. Sites (2, 3, 4) are similar in floristic composition with 15 species in each. Site 5 represents desert plain habitat and supports 14 species with different frequencies. The most common species located in the last site are *Asphodelus aestivus* (density = 33) and *Euphorbia retusa* (density = 24) (Table 3).

### 3.3. Phenological Aspects

**Figure 1:**
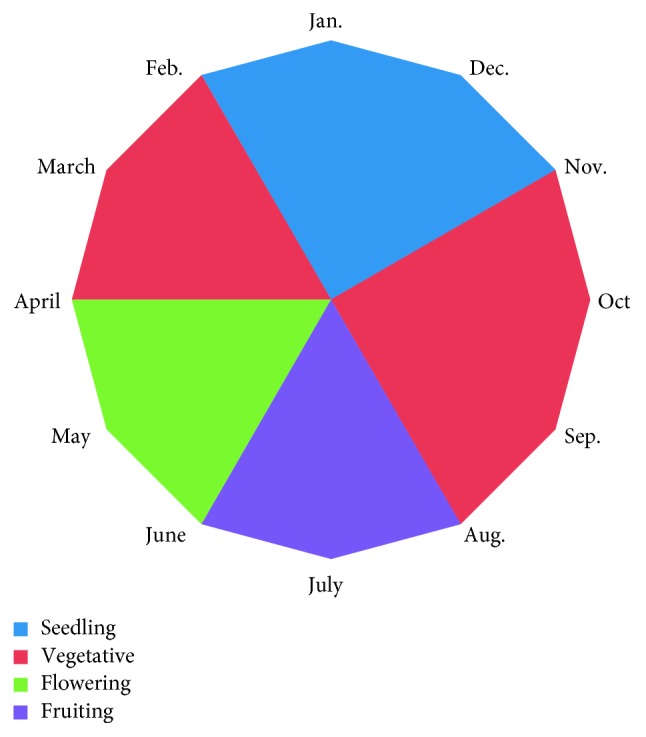
Phenological sequences of *Euphorbia retusa*.

Phenological sequences of *Euphorbia retusa* are summarized in Figure 1. The most common growth stage around the year is the vegetative stage which started in the beginning of February to April, and the plant becomes vegetative and dormant from August to November. The seedling stage started at the beginning of December to February, whereas flowering and fruiting stages started from April to August, respectively.

The general trends and ranking of vegetation are similar to the prevailing type in the western Mediterranean coast of Egypt where there are five main aliens predominate, namely, *Haloxylon scoparium*, *Asphodelus aestivum*, *Atriplex halimus*, *Thymelaea hirsuta*, and *Salsola tetrandra* [18]. Vegetation is mostly represented with shrubs and undershrubs or perennial herbs. Phanerophytes are less represented except some individuals especially in catchment areas in wadi beds. The phenological aspects of our species are mostly represented with the vegetative stage whatever, before or after raping. This sequence is playing a very important role in chemical investigation studies.

### 3.4. Phytochemical Study

**Figure 2:**
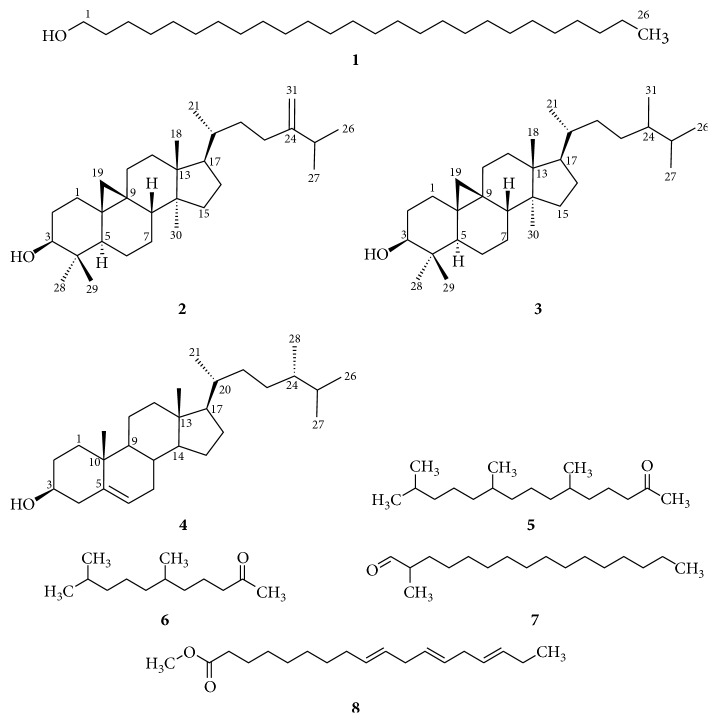
Structures of the obtained compounds **1–8** from *Euphorbia retusa*.

The importance of *Euphorbia retusa* as an interest source of diverse bioactive compounds served in treatment of diverse infectious diseases [12, 13] encouraged the authors herein to study the entire constituents of its organic extract. In accordance, an air-dried sample of the plant was applied to intensive extraction with methanol followed by concentration in vacuo, and the obtained dark green crude extract was then applied to a series of chromatographic purifications monitored by TLC and visualizing agents delivering five diverse compounds, namely, 1-hexacosanol (**1**), 3*β*-hydroxy-24-methylene-9,19-cyclolanostane (**2**), 3*β*-hydroxy-9,19-cyclolanostane (**3**), 3*β*,24*S*-ergost-5-en-ol (**4**), and methyllinoleate (Figure 2). Alternatively, analysis of the individual different unpolar fractions by GC-MS revealed the existence of further nine compounds as listed in Table 2 utilizing alkane internal standards (C8–C22) during the analyses for the determination of linear retention indices (LRIs) and assay performance.

#### 3.4.1. 1-Hexacosanol

As colorless needles, compound **1** was obtained as an extremely unpolar major substance from the fast fractions during a silica gel column eluted with cyclohexane. It showed no UV activity on TLC; however, it has been detected as violet and turned later as blue on spraying with anisaldehyde/sulphuric acid. The molecular weight of **1** was deduced as 382 Dalton according to EI-MS, displaying a further ion peak at *m*/*z* 364 due to the expulsion of a water molecule. The HREI-MS of **1** proved the corresponding molecular formula as C_26_H_54_O, displaying no degree of unsaturations, and hence, an aliphatic open chain of *sp*^3^ hybridization nature for **1** was concluded.

The ^1^H NMR spectrum exhibited a triplet oxymethylene signal at *δ* 3.62, and its corresponding carbon signal was confirmed at *δ*_*C*_ 63.1. Additional quartet methylene signal was visible at *δ*_*H*_ 1.54 and its corresponding carbon value at *δ*_*C*_ 32.9. This was in addition to a broad multiplet signal with integration of 36 protons being for 18 methylene groups, and their corresponding carbons were visible in the range of *δ* 32.0–22.8, terminated by a triplet methyl group at *δ* 0.85 (*δ*_*C*_ 14.2). Based on the revealed chromatographic and spectroscopic features, compound **1** was confirmed as 1-hexacosanol.

Biologically, *n*-hexacosanol, as long-chain fatty alcohol, was reported to promote the maturation of central neurons, and hence, it is known as neurotrophic factor [19], reducing the neuronal damage induced by the neurotoxin, kainic acid [20]. Alternatively, hexacosanol showed a stimulation of insulin secretion in vivo and in vitro, inducing a reduction of the insulin response to an intravenous glucose tolerance test with a consequent increase in hyperglycaemia, and hence, it has antidiabetic effects [21, 22].

#### 3.4.2. 24-Methylenecycloartanol

As further colorless needles, compound **2** was obtained as the most predominant substance in the plant's extract with additional low polarity. The compound displayed no UV activity on TLC as well, while it was detected as pink on spraying with anisaldehyde/sulphuric acid and turned later as violet, referring most likely to a terpenoidal moiety [23]. According to the EI-MS, compound **2** exhibited a molecular ion peak at *m*/*z* 440 followed by expulsion of the methyl group to afford a fragment ion peak at *m*/*z* 425. The molecular weight of **2** was further established as 440 Dalton by ESI-MS showing a *quasi*-ion peak at *m*/*z* 463 [M + Na]^+^ and the corresponding molecular formula as C_31_H_52_O, bearing six DBE.

**Figure 3:**
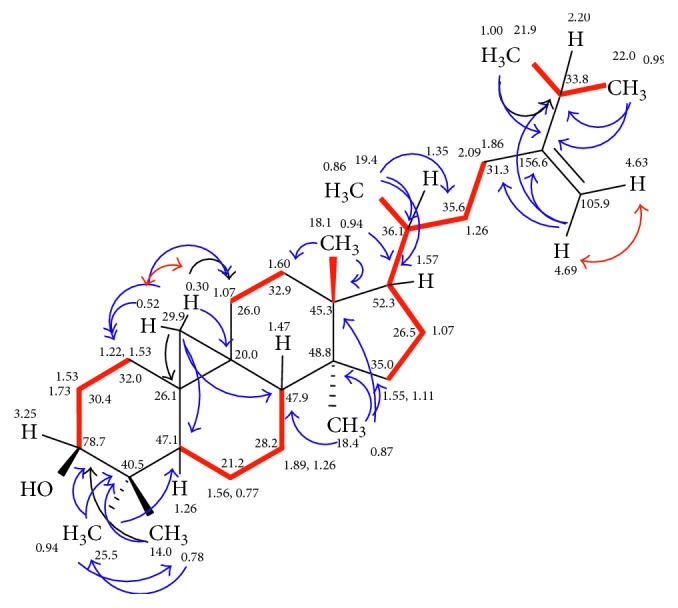
H,H COSY (▬, ↔) and selected and HMBC (→) correlations of 24-methylenecycloartanol (**2**).

The ^1^H NMR spectrum displayed two resonating signals with a tiny coupling constant (*J*∼1.7) being for an exomethylene group, whereas its carbon was deduced at *δ* 105.9 according to HMQC experiment and the complementary quaternary carbon at *δ* 156.6. Additionally, a multiplet signal for an oxymethine was at *δ* 3.25 and its carbon at *δ* 78.7. Furthermore, two highly upfield shifted doublet signals (each of *J*∼4.2) were observed at *δ* 0.52 and 0.30, corresponding to the highly strained methylene group (*δ*_*C*_: 29.9) existence in cyclopentane moiety. An additional NMR study (^1^H, ^13^C, HMQC, Table 4 and Figure 3) of compound **2** confirmed the existence of further seven methyls (classified into four singlet and three doublet signals), twelve methylenes (among them those of exomethylene and cyclopentane one), six methines (including the 3-hydroxy-methine one), and six quaternary carbons among them the olefinic one (156.6) as matched with the revealed molecular formula. An intensive study of compound **2** on the bases of H,H COSY, and HMBC experiments (Figure 3) confirmed its structure definitely as 3*β*-hydroxy-24-methylene-9,19-cyclolanostane.

Compound **2** was reported as a constituent of rice bran oil and woods from *Tristania conferta*, *Angophora subvelutina*, and *Cephalosphaera usambarensis* [24, 25]. It was reported as well from *Populus*, *Solanum*, *Betula*, and *Euphorbia*. The α-form of compound **2** was alternatively obtained from *Larix kaempferi* [26]. Three esterified tetracyclic triterpenes of compound **2** were recently reported from the dichloromethane extract of the roots of *E. retusa*: 24-methylenecycloartanyl formate, 24-methylenecycloartanyl 2′*E*,4′*E*-decadienoate, and tirucalla-7,24-dien-3-yl 2′*E*,4′*E-*decadienoate [27]. Biologically, compound **2** was reported to prevent diet-induced obesity in the liver through GIP-dependent and GIP-independent mechanisms [28]. Compound **2** was additionally reported to exhibit anti-inflammatory activity [29]. The compound is useful also as lipase inhibitors, fat absorption inhibitors, GIP increase inhibitors, postprandial hyperglycemia inhibitors, and antiobesity agents [30] and exhibited a moderate cytotoxicity against the cancer cell lines MDA-MB48 and MCF-7 [31].

#### 3.4.3. 3β-Hydroxy-9,19-cyclolanostane; Cyclolaudanol

**Figure 4:**
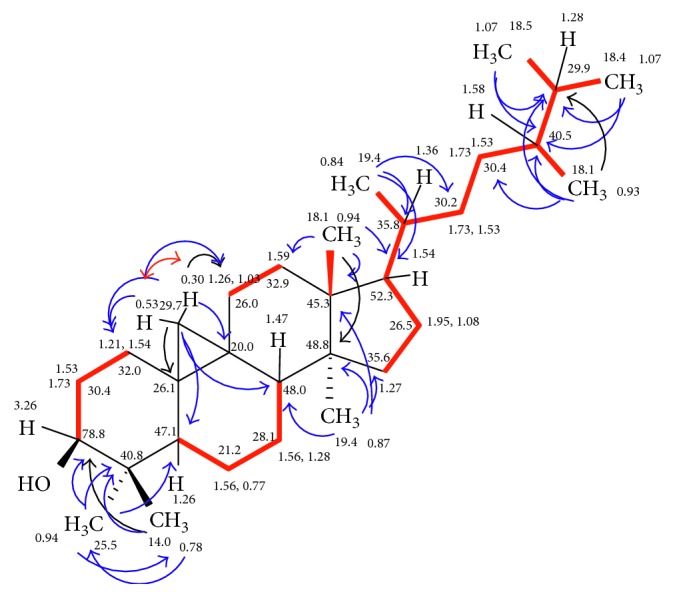
H,H COSY (▬, ↔) and selected and HMBC (→) correlations of 3*β*-hydroxy-9,19-cyclolanostane (**3**).

As closely related to compound **2**, with identical chromatographic properties, compound **3** was obtained exhibiting a tiny higher polarity than compound **2**, indicating a further triterpene system. According to the ESI-MS, the molecular weight of **3** was deduced as 442 Dalton due to the existence of two *quasi*-ion peaks at *m*/*z* 465 [M + Na]^+^ and 442 [M + H]^+^, with higher 2 amu than compound **2**. The corresponding molecular formula was subsequently established as C_31_H_54_O bearing five DBE (i.e., less double bonds than in compound **2**). Based on that, it clearly appeared that compound **3** is a hydrogenated analogue of compound **2**, and this was definitely confirmed from the ^1^HNMR spectrum, at where the olefinic protons of the exomethylene group in compound **2** disappeared, while a new doublet methyl visible at 0.93 was created. Detailed NMR study of compound **3** using 1 (^1^H, ^13^C) and 2D (H,H COSY, HMQC, and HMBC) (Table 4 and Figure 4) confirmed its structure as 3*β*-hydroxy-9,19-cyclolanostane [32].

Pharmaceuticals which contain cycloartanol, 24-methylcycloartanol, and/or 24-methylenecycloartanol in addition to highly unsaturated oils containing more than 3 double bonds have the capability to control the lipid metabolism in serum, and hence, they showed anticholesteremic effects [33].

#### 3.4.4. 3β,24*S*-Ergost-5-en-ol

As an alternative colorless solid, exhibiting no UV activity during TLC, compound **4** was afforded; nevertheless, it exhibited a violet coloration on spraying with anisaldehyde/sulphuric acid and turned later as blue, indicating a fatty acid or steroidal compound's nature. According to intensive spectroscopic study and comparison with corresponding literature, the structure of compound **4** was deduced as 3*β*,24*S*-Ergost-5-en-ol (Table 5). Compound **4** was reported previously as a constituent of rapeseed oil (*Brassica napa*), soybean oil (*Glycine max*), and wheat-germ oil (*Triticum* spp.) and found in virtually all plant oils [34–36].

### 3.5. Complementing Chromatographic and Spectroscopic Results for Compounds **1**–**4**

#### 3.5.1. 1-Hexacosanol (**1**)

C_26_H_54_O (382); colorless solid, non-UV absorbing (254 nm), and stained violet on spraying with anisaldehyde/sulphuric acid, turned later as blue. *R*_*f*_: 0.55 (CH_2_Cl_2_). ^1^H (300 MHz, CDCl_3_): *δ* 3.62 (t, *J* = 6.6 Hz, 2H), 1.54 (q, *J* = 6.8 Hz, 2H), 1.24 (m, 36H), 0.85 (t, *J* = 6.7 Hz, 3H). ^13^C NMR (125 MHz, CDCl_3_): *δ* 63.1 (CH_2_-1), 32.9 (CH_2_-2), 25.8 (CH_2_-3), 29.8-29.4 (CH_2_-4-23), 32.0 (CH_3_-24), 22.8 (CH_2_-25), 14.2 (CH_3_-26). EIMS (70 EV): *m*/*z* (%) 382 ([M]^+·^) (2), 364 ([M-H_2_O]^+^) (12), 336 (5), 280 (2), 265 (2), 237 (3), 223 (2), 209 (3), 195 (4), 181 (5), 167 (6), 153 (8), 139 (9), 125 (17), 111 (28), 97 (50), 83 (54), 69 (51), 57 (65), 43 (100). HREIMS: 382.4170 (calc. 382.4169 for C_26_H_54_O).

#### 3.5.2. 24-Methylenecycloartanol (**2**)

C_31_H_52_O (440); colorless solid, non-UV absorbing (254 nm), and stained pink on spraying with anisaldehyde/sulphuric acid, changed later to brown. *R*_*f*_: 0.50 (CH_2_Cl_2_). [α]^20^_D_ + 9.0 (c, 0.14 MeOH) (Lit: [α]_D_ + 43) [37]. ^1^H (300 MHz, CDCl_3_) and ^13^C NMR (125 MHz, CDCl_3_) (Table 4). (+)-ESI-MS: *m*/*z* (%) 463 ([M + Na]^+^). EIMS (70 EV): *m*/*z* (%) 440 ([M]^+·^) (13), 425 (15), 422 (18), 407 (24), 393 (16), 379 (15), 353 (9), 339 (7), 300 (18), 297 (9), 255 (11), 216 (15), 218 (10), 203 (20), 187 (19), 175 (28), 161 (25), 147 (30), 135 (34), 119 (37), 109 (42), 107 (50), 95 (64), 81 (55), 89 (100). HREIMS: 440.40738 (calc. 440.40736 for C_31_H_52_O).

#### 3.5.3. 3β-Hydroxy-9,19-cyclolanostane; Cyclolaudanol (**3**)

C_31_H_54_O (442); colorless solid, non-UV absorbing (254 nm), and stained pink on spraying with anisaldehyde/sulphuric acid and changed later to brown. *R*_*f*_: 0.40 (CH_2_Cl_2_). ^1^H (300 MHz, CDCl_3_) and ^13^C NMR (125 MHz, CDCl_3_) (Table 4). (+)-ESI-MS: *m*/*z* (%) 465 ([M + Na]^+^, 100), 443 ([M + H]^+^, 33). (+)-HRESI-MS: 465.40730 ([M + Na]^+^, calc. 465.40721 for C_31_H_54_ONa).

#### 3.5.4. 3β,24*S*-Ergost-5-en-ol (**4**)

C_28_H_48_O (400); colorless solid, UV-non-absorbing (254 nm), and stained blue on spraying with anisaldehyde/sulphuric acid. *R*_*f*_: 0.20 (CH_2_Cl_2_). ^1^H (300 MHz, CDCl_3_) and ^13^C NMR (125 MHz, CDCl_3_) (Table 5). EI-MS: *m*/*z* (%) 400.4 ([M]^+·^, 42), 382.4 ([M-H_2_O]^+^, 23), 367.4 (16), 315.3 (24), 289.3 (15), 271.2 (21), 255.2 (25), 231.2 (16), 213.2 (26), 199.2 (13), 159.1 (24), 145.1 (28), 133.1 (22), 107.1 (28), 95.1 (33), 81.1 (36), 71.1 (40), 69.1 (48), 57.1 (57), 55.1 (62).

### 3.6. Biological Activity

Antimicrobial activity testing of the crude extract of *Euphorbia retusa* was carried out against seven microorganisms using the agar diffusion technique with paper platelets (400 *μ*g per disk), exhibiting no activity against all test organisms: the Gram-negative bacteria *Pseudomonas* sp., *Shigella* sp., *Proteus* sp., and *Escherichia coli*, the Gram-positive bacteria *Staphylococcus aureus*, *Streptococcus pyogenes*, *Bacillus subtilis* ATCC6051, and *Streptomyces viridochromogenes* Tü 57, the fungi *Acremonium* sp. and *Mucor miehei* Tü 284, the yeast *Candida albicans*, and the green alga *Chlorella vulgaris*.

Alternatively, an antibacterial activity retesting of the crude extract of *Euphorbia retusa* in comparison with compounds (**1–4**) was carried out against Gram-positive bacteria (*Micrococcus luteus* DSMZ 1605, *Staphylococcus warneri* DSMZ 20036, and *Bacillus subtilis* DSMZ 704) and Gram-negative bacteria (*Pseudomonas agarici* DSMZ 11810 and *Escherichia coli* DSMZ 1058) in comparison with gentamicin (Table 6). Based on that, the plant extract exhibited weak activity against *Staphylococcus warneri* (8 mm) and *Escherichia coli* (7 mm), meanwhile compounds **1–4** displayed no antibacterial activity. Alternatively, the plant extract and the corresponding compounds were tested for in vitro cytotoxicity against the human cervix carcinoma cell line (KB-3-1) in comparison with griseofulvin. The results revealed that the plant extract and the afforded compounds have no cytotoxicity (Table 7).

## 4. Conclusion

The ecological study of *Euphorbia retusa* species in five selected sites in Sidi Barrani area, western coast of Egypt, was intensively carried out. Measuring of the density, frequency, and flowering sequences of *Euphorbia retusa* and its associated species was reported. Phytochemical study of the plant was visualized affording a broad range of diverse compounds, bearing mostly a rich abundance of carotenoids, fatty alcohol chains (e.g., 1-hexacosanol (**1**)), triterpenes (3*β*-hydroxy-24-methylene-9,19-cyclolanostane (**2**) and 3*β*-hydroxy-9,19-cyclolanostane (**3**)), sterols (3*β*,24*S*-Ergost-5-en-ol (**4**)), and essential fatty acids (EFAs) (e.g., linoleic acid, an omega 6 fatty acid). Analysis of the unpolar fractions using GC-MS detected the existence of numerous diverse oxygenated hydrocarbons. Biologically, the plant extract and the desired isolated compounds are inactive against Gram-positive and Gram-negative bacteria. Examination of the cytotoxicity of the plant extract along with its entire bioactive compounds confirmed its noncytotoxicity against the human cervix carcinoma cell line (KB-3-1).

## Figures and Tables

**Table 1:** Position, location, and habitats of 5 selected sites represent *Euphorbia retusa* species in Sidi Barrani area, western coast of Egypt.

Site	Coordinate	Habitat	Community type
1	31 32 20.8N, 025 42 31.2E	Plateau	*Thymelaea hirsuta*
2	31 28 19.4N, 025 40 49.4E	Desert plain	*Asphodelus aestivus*
3	31 29 25.0N, 025 42 04.8E	Plateau	*Lycium europaeum*
4	31 28 43.7N, 025 40 52.0E	Slopes	*Haloxylon scoparium*
5	31 29 34.5N, 025 40 27.8E	Desert plain	*Asphodelus aestivus*

**Table 2:** GC-MS results of different unpolar fractions obtained from *Euphorbia retusa* extract.

Name	LRI	Rt (min)	Relative abundance (%)	Mol. formula	Mol. weight
*n*-Dodecane	656.76	8.08	100	C_12_H_26_	170
Methyllaurate; methyldodecanoate	893.29	10.99	10	C_13_H_26_O_2_	214
6,10,14-Trimethyl-pentadecan-2-one (**5**)	1090.00	13.41	33	C_18_H_36_O	268
6,10-Dimethyl-undecan-2-one (**6**)	1093.25	13.45	57	C_13_H_26_O	198
2-Methyl-hexadecanal (**7**)	1132.27	13.93	82	C_17_H_34_O	254
Methylpalmitate; methylhexadecanoate	1135.52	13.97	50	C_17_H_34_O_2_	270
Methyl-9,12,15-octadecatreinoate (**8**)	1165.81	15.13	100	C_19_H_32_O_2_	292
Methylstearate; methyloctadecanoate	1177.37	15.26	26	C_19_H_38_O_2_	298
*n*-Heneicosane (**9**)	1252.25	17.35	11	C_21_H_44_	296

**Table 3:** A list of 30 species recorded in the study area. Species are represented with their relative density and frequency.

Species	Density					Frequency (%)
	Site 1	Site 2	Site 3	Site 4	Site 5	
Perennials						
*Asphodelus aestivus* Brot.	15	21	11	0	33	80
*Astragalus trigonus* DC.	0	0	0	7	0	20
*Centaurea calcitrapa* L.	5	8	0	0	0	40
*Citrullus colocynthis* (L.) Schrad.	0	0	0	0	6	20
*Convolvulus althaeoides* L.	0	0	0	17	0	20
*Deverra tortuosa* (Desf.) DC.	10	18	9	0	3	80
*Dipcadi erythraeum* Webb & Berthel.	0	0	2	0	0	20
*Echinops spinosus* L.	0	13	11	0	0	60
*Echiochilon fruticosum* Desf.	0	0	0	4	2	40
*Euphorbia retusa* Forssk.	11	5	12	19	24	100
*Farsetia aegyptia* Turra.	0	1	3	0	0	40
*Haloxylon scoparium* Pomel.	9	17	18	26	11	100
*Haplophyllum tuberculatum* (Forssk.) Juss.	0	0	0	5	0	20
*Lycium europaeum* L.	0	0	21	0	8	40
*Lygeum spartum* Loefl. ex L.	0	0	0	0	1	20
*Marrubium vulgare* L.	6	2	0	0	0	40
*Peganum harmala* L.	0	0	0	4	2	40
*Plantago albicans* L.	3	0	5	0	0	40
*Salvia lanigera* Poir.	0	2	0	5	0	20
*Scorzonera undulata* Vahl.	6	2	0	0	0	40
*Thymelaea hirsuta* (L.) Endl.	29	11	4	8	3	100
*Zygophyllum album* L.f.	6	0	4	4	7	80
Annuals						
*Cakile maritima* Scop.	0	0	1	1	0	40
*Carduus pycnocephalus* L.	1	1	0	1	0	60
*Cutandia memphitica* (Spreng.) Benth.	0	0	0	0	1	20
*Filago desertorum* Pomel.	0	0	1	0	0	20
*Heliotropium supinum* L.	0	1	0	0	1	40
*Ifloga spicata* (Forssk.) Sch.Bip.	0	0	1	1	0	40
*Lappula spinocarpos* (Forssk.) Asch. ex Kuntze.	1	1	0	0	0	40
*Medicago intertexta* (L.) Mill.	1	1	1	1	1	100
Total no. of plants	13	15	15	15	14	—

**Table 4:** ^13^C (125 MHz) and ^1^H (600 MHz) NMR data of compounds **2** and **3** in CDCl_3_.

Number	**2**		**3**	
	*δ*_*C*_	*δ*_*H*_ (*J*_Hz_)	*δ*_*C*_	*δ*_*H*_ (*J*_Hz_)
1	32.0	1.53 (m), 1.22 (m)	32.0	1.53 (m), 1.21 (m)
2	30.4	1.53 (m), 1.73 (m)	30.4	1.53 (m), 1.73 (m)
3	78.7	3.25 (m)	78.8	3.26 (m)
4	40.5	—	40.5	—
5	47.1	1.26 (m)	47.1	1.26 (m)
6	21.2	1.56 (m), 0.77 (m)	21.2	1.56 (m), 0.77 (m)
7	28.2	1.89 (m), 1.26 (m)	28.1	1.89 (m), 1.26 (m)
8	47.8	1.47 (m)	48.0	1.47 (m)
9	20.0	—	20.0	—
10	26.1	—	26.1	—
11	26.0	1.07 (m)	26.0	1.26, 1.03 (m)
12	32.9	1.60 (m)	32.9	1.59 (m)
13	45.3	—	45.3	—
14	48.8	—	48.8	—
15	35.0	1.55 (m), 1.11 (m)	35.6	1.27 (m)
16	26.5	1.07 (m)	26.5	1.95 (m), 1.8 (m)
17	52.3	1.57 (m)	52.3	1.54 (m)
18	18.1	0.94 (s)	18.1	0.94 (s)
19	29.9	0.52 (d, 4.2), 0.30 (d, 4.2)	29.7	0.53 (d, 4.2), 0.30 (d, 4.2)
20	36.1	1.35 (m)	35.8	1.36 (m)
21	19.4	0.86 (d, 6.8)	19.4	0.84 (d, 6.8)
22	35.6	1.26 (m)	30.2	1.73 (m), 1.53 (m)
23	31.3	2.09 (m), 1.86 (m)	30.4	1.73 (m), 1.53 (m)
24	156.6	—	40.5	1.58 (m)
25	33.8	2.20 (m)	29.9	1.28 (m)
26	21.9	1.00 (d, 6.8)	18.5	1.07 (d, 6.8)
27	22.0	0.99 (d, 6.8)	18.4	1.07 (d, 6.8)
28	25.5	0.94 (s)	25.5	0.94 (s)
29	14.0	0.78 (s)	14.0	0.78 (s)
30	18.4	0.87 (s)	19.4	0.87 (s)
31	105.9	4.69 (d, 1.7), 4.63 (d, 1.7)	18.1	0.93 (d, 6.8)

**Table 5:** ^13^C (125 MHz, CDCl_3_) and ^1^H NMR (300 MHz) data of 3*β*,24*R*-Ergost-5-en-ol (**4**).

Number	*δ*_*C*_ (125 MHz)	*δ*_*H*_ (300 MHz)	Number	*δ*_*C*_ (125 MHz)	*δ*_*H*_ (300 MHz)
1	37.2	1.80 (m), 1.04 (m)	15	24.2	1.54 (m), 1.04 (m)
2	31.5	1.80 (m), 1.47 (m)	16	24.2	1.54 (m), 1.04 (m)
3	71.6	3.48 (m)	17	56.1	1.07 (m)
4	42.1	2.24 (m), 2.19 (m)	18	11.8	0.64 (s)
5	140.5	—	19	19.3	0.97(s)
6	121.5	5.41 (dt, 4.7, 2.2)	20	36.1	1.32 (m)
7	31.8	1.95 (m)	21	18.8	0.88 (d, 6.7)
8	31.8	1.40 (m)	22	33.6	1.37 (m), 0.92 (m)
9	50.0	0.91 (m)	23	31.8	1.92 (m)
10	36.4	—	24	39.0	1.16 (m)
11	21.0	1.46 (m), 1.43 (m)	25	31.4	1.54 (m)
12	39.7	1.97 (m), 1.11 (m)	26	17.6	0.75 (d, 6.7)
13	42.2	—	27	20.5	0.82 (d, 6.6)
14	56.6	0.96 (m)	28	15.4	0.74 (d, 6.8)

**Table 6:** Antimicrobial activities of *Euphorbia retusa* extract and pure compounds (**1–4**) in the agar diffusion test (mm diameter).

Ext./compound	Conc. (mg/mL)	EC^a^	BS^b^	Psa^c^	Ml^d^	Stw^e^
*Euphorbia retusa* extract	40.0	7	—	—	—	8
**1**	1.0	—	—	—	—	—
**2**	1.0	—	—	—	—	—
**3**	1.0	—	—	—	—	—
**4**	1.0	—	—	—	—	—
Gentamicin	0.5	24	24	25	24	19

^a^ *E. coli* DSMZ 1058; ^b^*Bacillus subtilis* DSMZ 704; ^c^*Pseudomonas agarici* DSMZ 11810; ^d^*Micrococcus luteus* DSMZ 1605; ^e^*Staphylococcus warneri* DSMZ 20036; (—) = no activity.

**Table 7:** In vitro cytotoxicity of *Euphorbia retusa* extract and compounds **1–4** against KB-3-1 cell line.

Compound/extract	IC_50_ KB-3-1 (0.8–7.8 E^−04^ mg/mL)
*Euphorbia retusa* extract	>0.1
**1**	—
**2**	—
**3**	—
**4**	—
Griseofulvin	19
